# FEM Investigation of the Stress Distribution over Mandibular Bone Due to Screwed Overdenture Positioned on Dental Implants

**DOI:** 10.3390/ma11091512

**Published:** 2018-08-23

**Authors:** Marco Cicciù, Gabriele Cervino, Dario Milone, Giacomo Risitano

**Affiliations:** 1 Department of Biomedical and Dental Sciences and Morphological and Functional Imaging, Messina University, 98100 Messina, Italy; gcervino@unime.it; 2 Department of Engineering, Messina University, 98100 Messina, Italy; dario.milone94@gmail.com (D.M.); grisitano@unime.it (G.R.)

**Keywords:** masticatory loads, stress distribution, FEM

## Abstract

The objective of the present investigation was to evaluate how dental implant positioning can influence the masticatory stress distribution over screwed mandibular prosthodontics restoration and over the surrounding bone tissue. Moreover, the dental implant components and overdenture bar strengths under masticatory cycles have been investigated in order to evaluate possible screw and prosthesis breakage. A “virtual jaw” model and 3D dental implant were reproduced to realise finite element analysis in order to underline the parameters and the mechanical characteristics of the bone and of the dental implants connected to the overdenture bar. The distribution of a nonspecific chewing phase, analysing the overall load on the fixtures of the lower jaw, was performed. The study investigating frontal and horizontal planes and vertical directions of occlusal forces showed how position and perspective of fixtures strongly influenced the stress distribution and the consequent jawbone tissue remodelling. Prostheses elements such as cantilever, passing screws, and dental implants are strictly related to the correct selection of dental implant position. This study suggested a virtual method to guide the surgeon in the choice of implant number, position, diameter, and length, and cantilever length and shape, and to evaluate the prospective stress distribution of chewing strengths for a correct prosthesis rehabilitation.

## 1. Introduction

The modification of the bone tissue related to mechanical influence is a condition that has been analysed since the middle of the 19th century [1]. The alveolar bone is continuously subjected to the action of axial and lateral forces during masticatory cycles, and this physiological event determines deformation or strain over the periodontal tissues distributed over teeth, periodontal ligament, and alveolar bone. Bone tissue growth or pathological events occur due to remodelling processes. This dynamic balance is influenced by two dynamic mechanisms: one of deposition due to osteoblast and the other of resorption due to osteoclast actions [1,2,3,4].

In different clinical conditions and especially after the loss of teeth, bone undergoes a process of progressive atrophy. Tallgren observed that the greatest proportion of the alveolar bone loss occurs in the first year [2]. Moreover, how the alveolar bone undergoes physiological turnover as is seen with other bones has been evaluated, and during life it is involved in significant remodelling during tooth movement, masticatory loads, and other external stimuli like trauma or orthodontic tension [3,4].

The alveolar bone volume and maintenance is related to the tooth health, such that following tooth extraction it is slowly resorbed down to the base of the jawbone. In the case of complete tooth loss, there is continuous bone resorption; this can lead to deep atrophy of the jawbones, which can present significant clinical disadvantages for dental implant positioning and construction of dental prostheses [2,4]. All these parameters have to be evaluated before approaching the prosthodontics dental implant rehabilitation of an edentulous patient in order to restore long-term oral function and aesthetics and to achieve the aimed clinical success. Accordingly, the primary objective of the present investigation is to evaluate the changes in facial bone due to the masticatory load by applying anatomical information through the classification of edentulous jaws described by Cawood and Howell and other data presented in the recent literature about stress distribution over the jaws and over dental implant structural components [4,5,6,7,8,9].

Dental implant rehabilitation for edentulous jaws and mandible is today considered a predictable, safe, and daily technique for giving patients new aesthetics and function. Screwed overdenture prosthetic restoration over dental implants and the use of a bar for removable prosthodontics can both be considered valid choices for the treatment of an edentulous patient with atrophy of the jaw [4,5,9].

In case of a dental implant screwed prosthesis, the main problem could be the fracture of the cantilever prosthesis or fracture of passant screw and consequent peri-implant bone loss with inflammation of peri-implant hard and soft tissue [6,8,10]. The posterior cantilever break is a common problem that usually does not occur immediately but after several masticatory cycles leading to prosthodontics component wear [5,6,7,8,9,10].

The principal aim of this study is analysis via the finite element method (FEM) of a dental implant placed over mandibular bone, recreating different conditions of stress and load with a dental overdenture bar prosthesis. For considering axial load, the masticatory load recorded on the mandibular posterior area was about 700–1000 Newtons [11,12,13,14,15]. In the treatment of the edentulous mandible, dental implants are generally positioned between the mental foramina to avoid injuring the inferior alveolar nerve [16,17,18,19,20]. A secondary aim of the present study was to perform an FEM comparative analysis of the strength distribution in the lower jaw related to implant position, evaluating the mandible bone stress. FEM analyses were performed to study the difference in the variation of the load for various dental implant positions and numbers.

## 2. Material and Methods

The study evaluates the distribution of stresses on a dental implant overdenture prosthesis in order to evaluate the possible failure related to the fracture of the biomechanical system.

**Figure 1:**
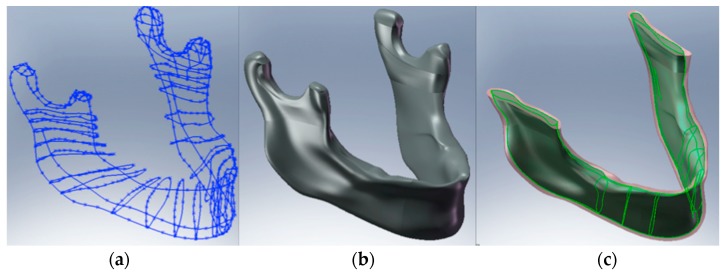
(**a**) Mandible drawing; (**b**) CAD model of the mandible, (**c**) model of cortical and midollar bone tissue areas.

**Figure 2:**
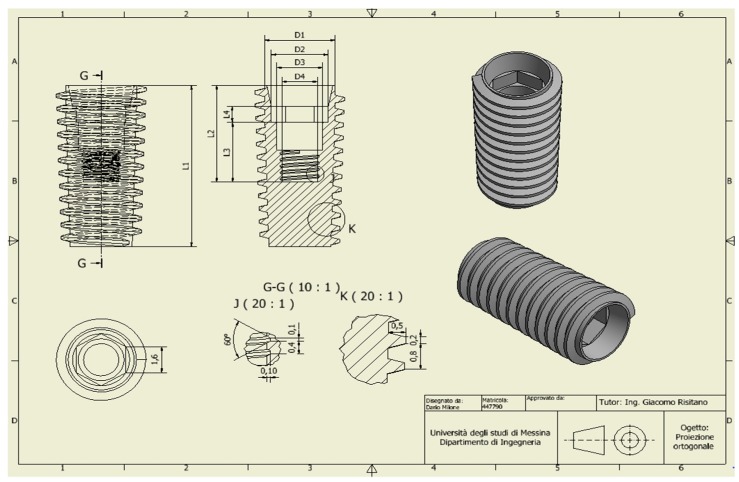
Dimensions of the fixture (Osstem^®^ TSIII) involved in the study.

**Figure 3:**
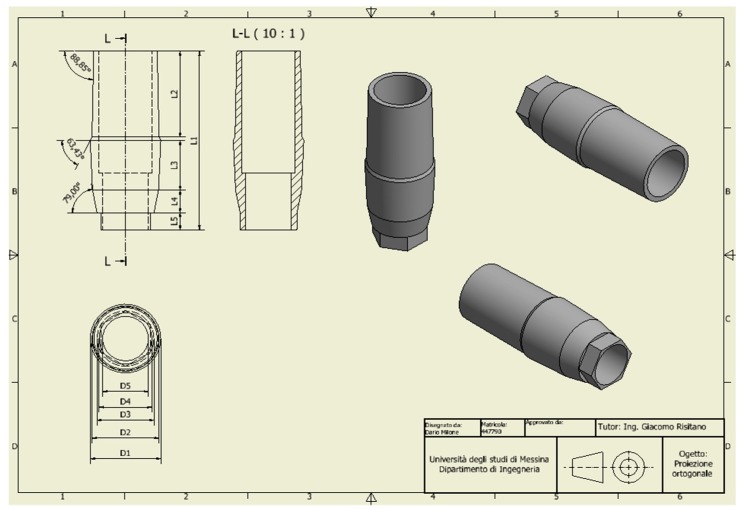
Dental implant abutment chosen accordingly with the connection system of the implant selected for the study.

**Figure 4:**
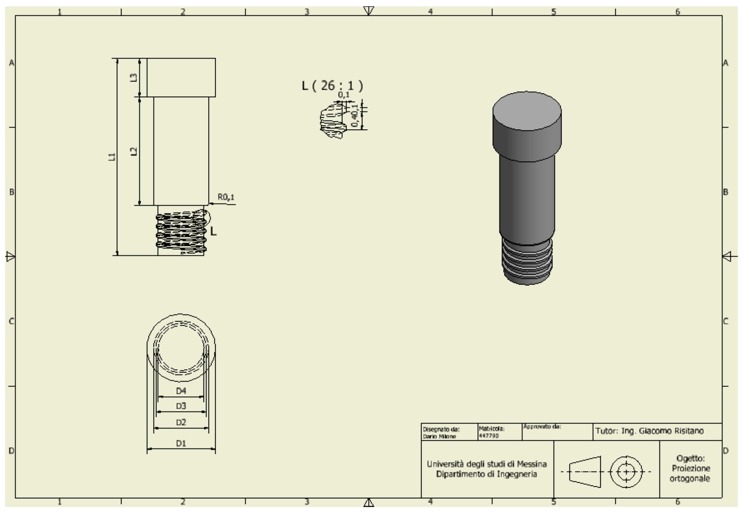
A particular drawing of the passant screw selected for the abutment–fixture–bar connection.

For the analysis, a CAD model of the skull and specifically of the jaw was created which perfectly reproduced the human mandible anatomy—the cortical and marrow bone—using SolidWorks^®^ (Dassaul Systeme, Waltham, MA, USA) (Figure 1). A computed tomography of a mandible was firstly elaborated from an edentulous skull anatomical human model and then recreated to produce a three-dimensional model. Then, the Osstem^®^-Micerium^®^, (Micerium, Genova, Italy) dental implant CAD virtual model Ø 4.0/4.5 mm 10.0 mm in length and characterised by grade 4 titanium alloy was created using engineering drawings of the implant by SolidWorks^®^ (Figure 2, Figure 3 and Figure 4).

The model of the force distribution was achieved using Matlab^®^ (influenced by dental implant positions) over dental implants during a generic chewing cycle [4].

Afterwards, Ansys Worchbench^®^ was used to perform finite element analysis (FEA) on the mandible–fixture system and showed the stresses and deformation between bone and implant.

Prior to studying the mechanical forces applied to the model, it is important to keep in mind that bone characteristics are connected with individual physical features and so there is high variability; consequently, the bone values and parameters were chosen from literary references [5,7,14,15,16,17,18].

**Figure 5:**
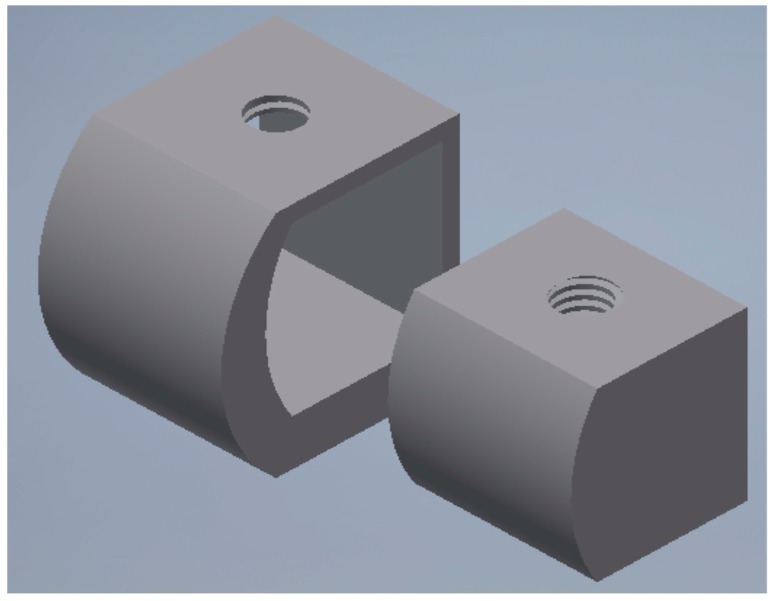
A recreation of mandibular bone. External cortical bone and internal midollar bone.

**Figure 6:**
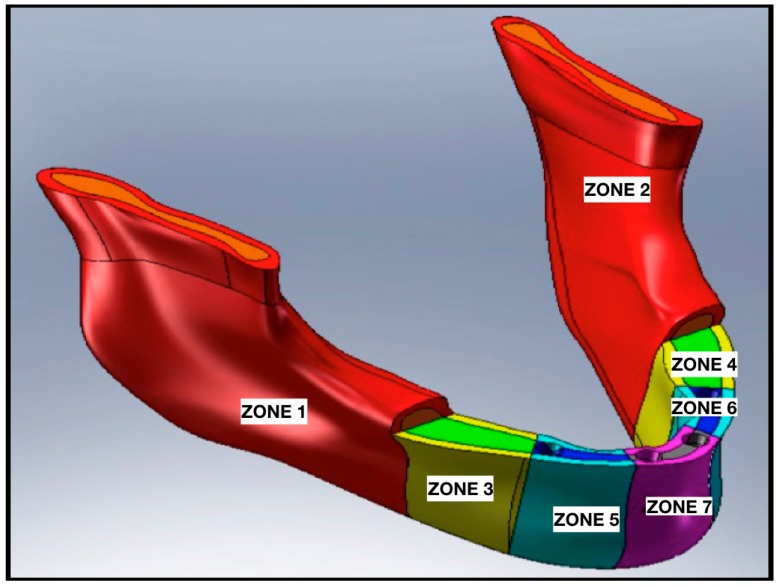
Mandibular model sectioned into different load areas and divided also into cortical and midollar bone, accordingly with literature data.

Accordingly with the literature data, the edentulous mandibular bone was firstly qualified into cortical and midollar (Figure 5) and then divided into seven different masticatory areas (Figure 6). Table 1 shows the values for each area also in cortical and marrowbone [2,3,4,5] and the mechanical characteristics of the titanium alloy used for the screws. The effect of loading strengths over dental implant elements and peri-implant bone can be recorded by applying the equivalent stress system evaluation (von Mises stress), expressed in Megapascals (MPa). The difference in tension distribution is generally highlighted by different colors and red is the maximum stress [1,4].

Axial loads are difficult to characterise because are influenced by different factors like the subject’s bio-individuality, the different muscle systems, and the curve and the angles of the upper and lower jaw and the teeth occlusal surfaces. So, to elaborate axial loads, the following conditions applied in Matlab^®^ were used:

- Analysis was performed on sagittal and axial planes.
- Occlusion over all transversal planes.
- Load of 200 N for anterior area, 600 N for premolars, and 800 N for molars.
- Only a static load condition has been studied.

**Figure 7:**
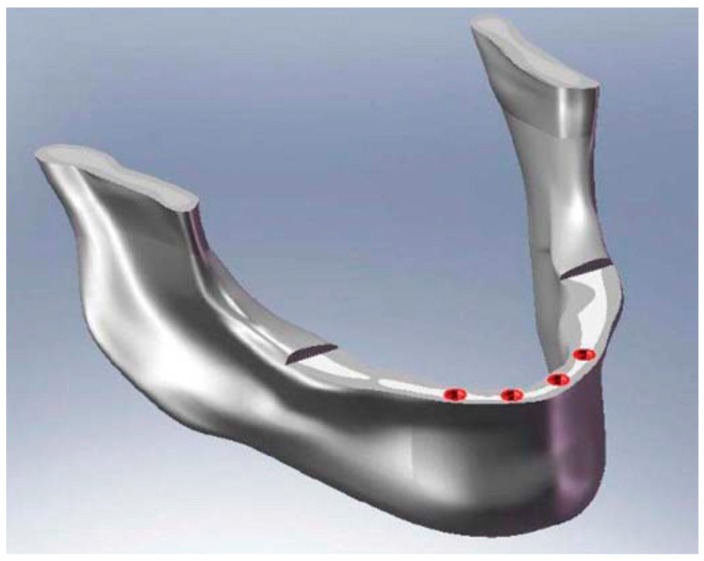
A mandibular 3D model of dental implant positioning in the interforamina position. This is a simple example of the ideal position of dental implants for overdenture bar prosthetic restoration.

**Figure 8:**
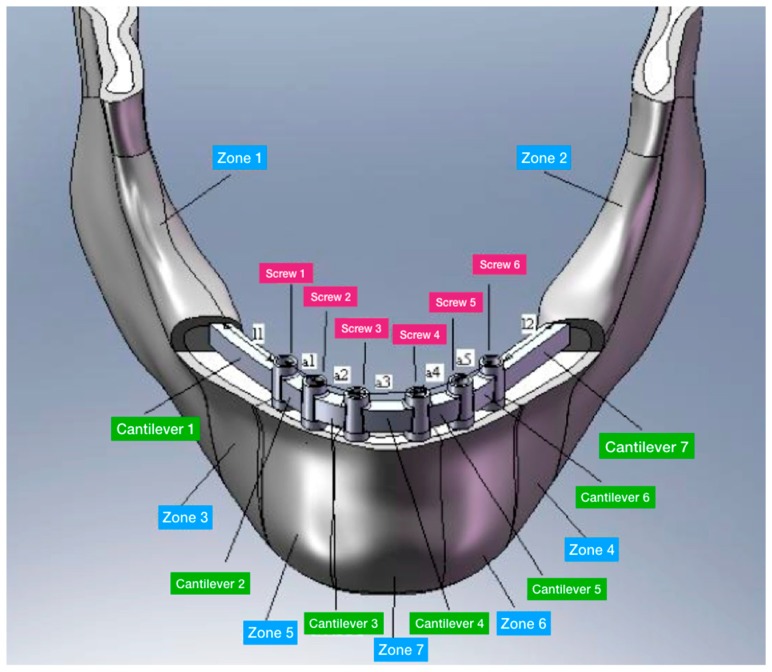
3D model with the prosthesis structure: l1 and l2 represent the cantilever; a1, a2, a3, a4 and a5 represent the spaces between the dental implants.

Four trials with different dental implant positions were performed using FEA, using as variables for each analysis the dental implants number and placement positions, but maintaining the same diameter as constant. As an example, Figure 7 shows the positions of the hole screw for one of the trials. The distance of the dental implant was chosen accordingly with the data reported in the literature, as shown in Table 2 and Figure 8.

Trials 1 and 2 were performed with six dental implants supporting a Toronto prosthesis. The difference between the two trials is that the cantilever length was longer in the first trial than in the second.

Trials 3 and 4 were performed with an ideal 4 dental fixture placed supporting an overdenture screwed prosthesis. Trial 3 and Trial 4 differed in the length of the prosthesis cantilever and of the dental implant placed in the lateral (premolar) area.

**Figure 9:**
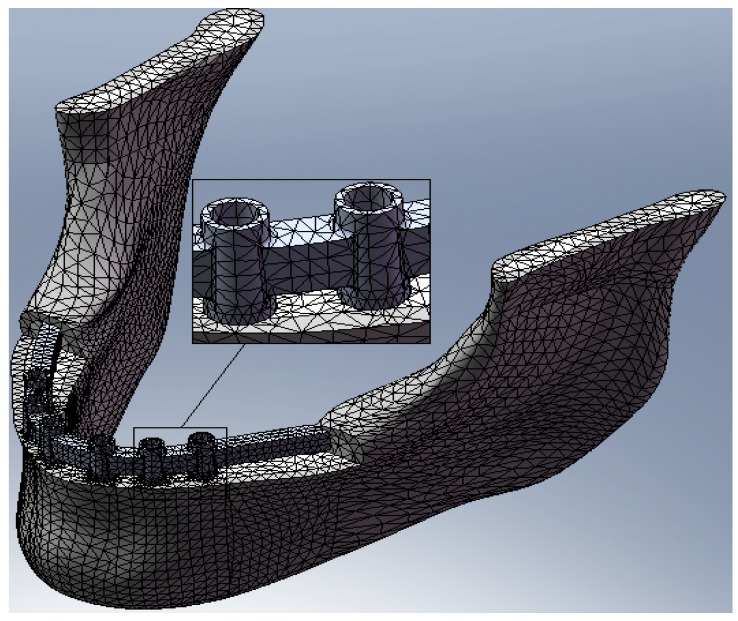
Sample of the mesh of the bone mandible, of the bar, and of the fixture placed.

The discretisation of the domain in this type of model is very complex because the simultaneous presence of several screws determines a very high number of elements. Therefore, it is necessary to reach a compromise between reduction of the number of elements and quality of the discrete model. Then, convergence analysis was conducted in order to verify which was the minimum dimension of elements below which the result of the FEM analysis remained unchanged. Table 3 shows the values used for the dimensions of the elements in the various components of the biomechanical system. The elements used are tetrahedr (SOLID 187). Figure 9 shows a sample of the mesh of the bone mandible, of the bar, and of the fixture placed.

The constraints between the various parts of the model have thus been modeled as “non-penetration” with the exception of the following:

- joint constraint between the overlapping faces of the bone components;
- joint constraint between the overlapping faces between fixture and connection screw;
- joint constraint between the threading of the fixture and the bone.

Finally, the interlocking constraints are applied to the extremity of the mandible near the joint. In this way, the constraint is sufficiently far from the area involved in the study and does not directly influence the results.

FEM analysis was performed for each trial. Static evaluation related to dental implant position was obtained.

## 3. Results

Simulating the stress and the masticatory cycles over the structures involved in the study, the analyses reported different data.

From the data analysis, it is evident that Trial 3 has average Von Mises stress values much higher than the other three cases. This is certainly due to the extreme length of the cantilever.

From a first analysis it turns out that the models with six screws (Trials 1 and 2) have better overall behaviour than does the one with four screws (Trial 4). Analysing this more in detail, it can be seen that this advantage is less pronounced for the bone.

Despite the results, it can be said that the difference between the values found is not as clear as could be expected. For this reason it can be assumed that the relative reduction of stress does not justify the use of a six-screw system for the following reasons:

- The use of the six-screw implant is exclusively available for those patients with a jaw size that facilitates the installation of screws in the posterior part of the dental arch.
- The possibility of installation of screws in the area of the molars greatly increases the risk of positioning them incorrectly, given the reduced space which is available to the implant surgeon. Recall that an incorrect positioning of the prosthesis-supporting abutments almost always generates a failure of the implant and, therefore, it is obvious that great care is needed when evaluating the six-screw solution as optimal.
- The use of two extra screws leads to an increase in the cost of the installation that cannot be neglected, especially if the advantages obtainable are not so clear.

**Figure 10:**
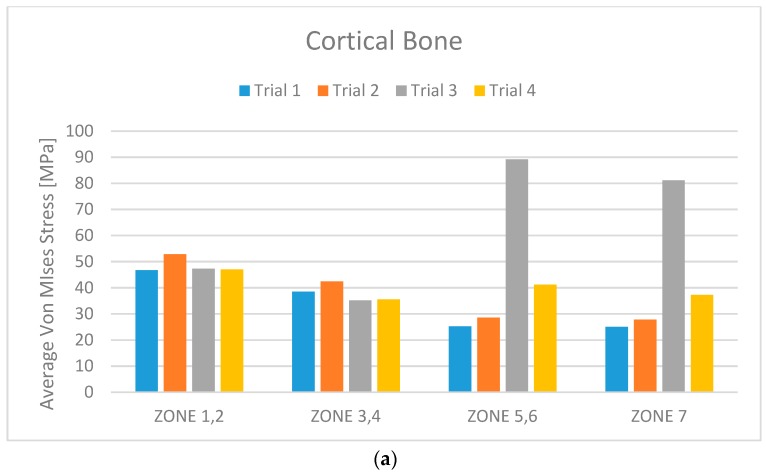

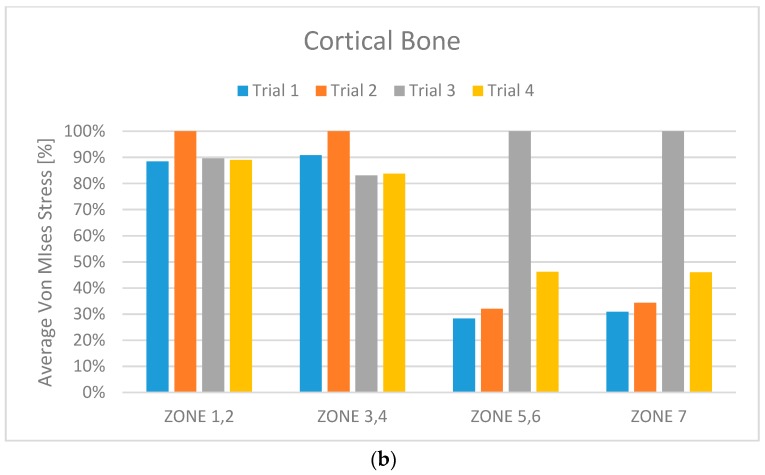
(**a**) Cortical bone stress distribution for each trial and for the different zones; (**b**) Cortical bone perceptual average Von Mises stress for each trial and for the different zones.

**Figure 11:**
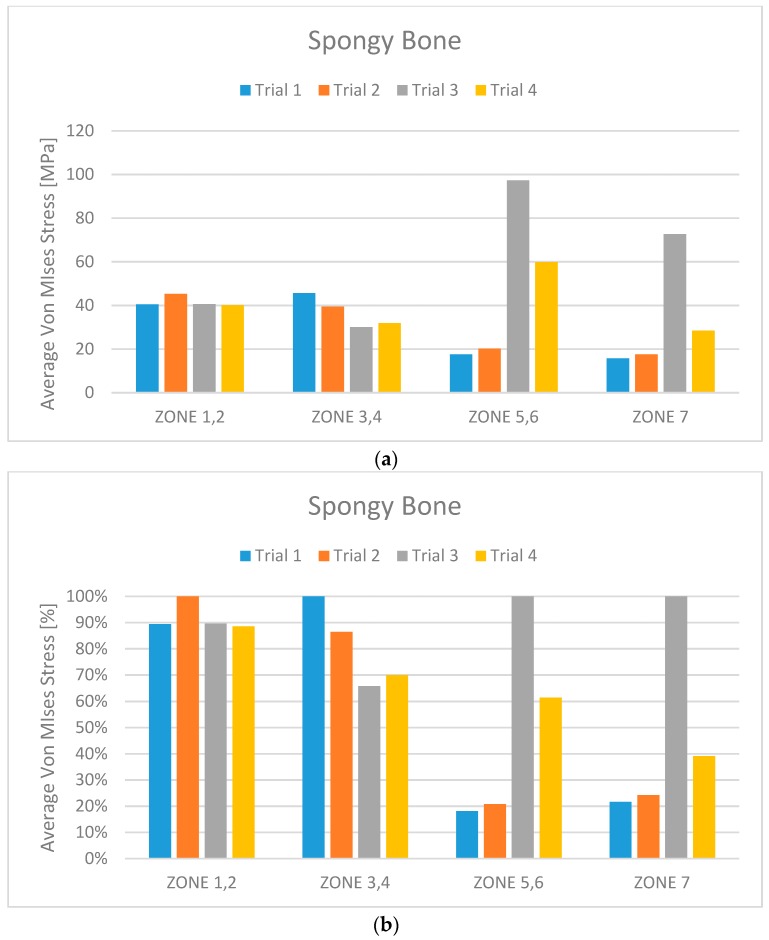
(**a**) Midollar bone stress maximum value for each trial and for the different zones; (**b**) Midollar bone percentage average Von Mises stress for each trial and for the different zones.

**Figure 12:**
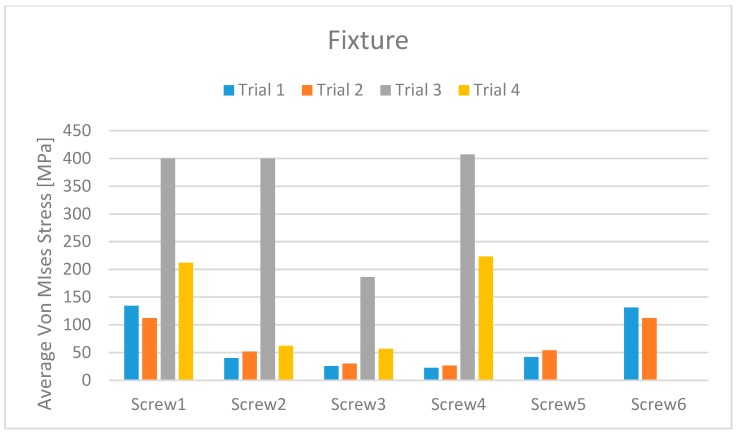
Dental implant stress maximum value for each trial and for the different screws.

**Figure 13:**
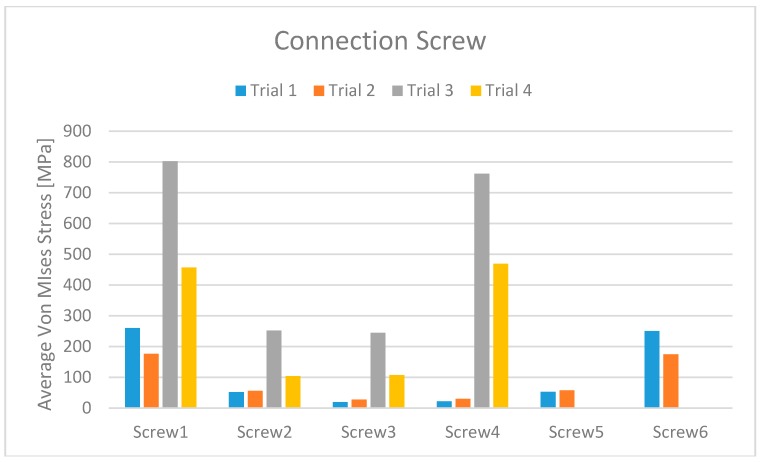
Passant screw stress maximum value for each trial and for the different fixtures.

**Figure 14:**
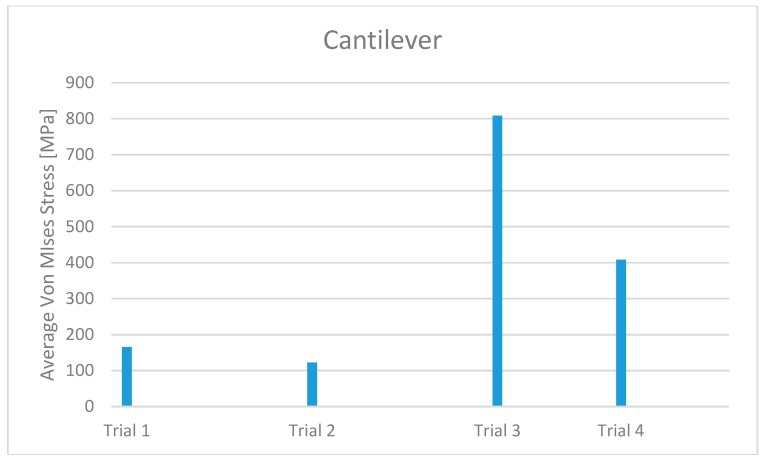
Cantilever stress maximum value for each trial and for the different zones.

**Figure 15:**
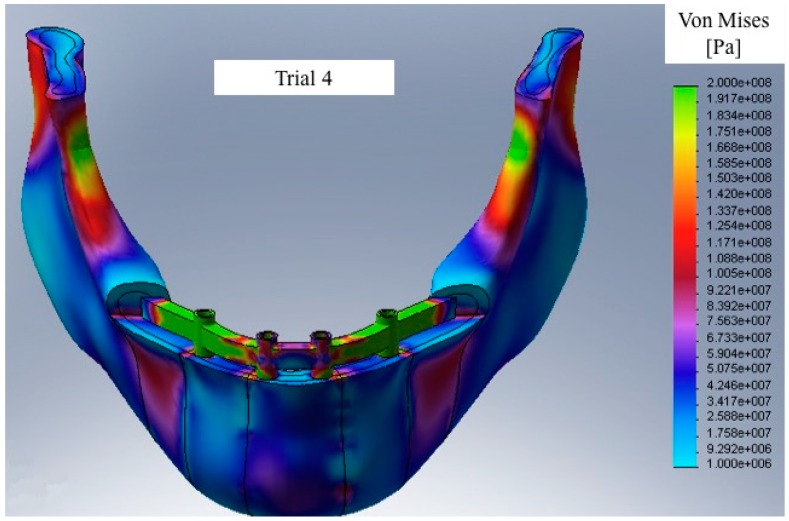
Sample of Von Mises equivalent stress vision with the model under stress in Trial 4.

Comparing Trial 1 and Trial 2, there are no major differences. In fact, Trial 1 has lower average stress on the bone with higher stress values on the prosthetic structure. Instead, at the expense of higher bone values, Trial 2 has less stress on the screws and the cantilever. The four-screw implant is the one that has the most suitable characteristics for installation on edentulous patients (Figure 10, Figure 11, Figure 12, Figure 13, Figure 14 and Figure 15).

## 4. Discussion

Today, the literature trend is to investigate dental implant failure or bone loss around dental implants, highlighting only the ratio between distal cantilever and length of the basis of the prosthesis by using stress testing and parametric analysis [11,12,13]. Therefore, the current tendency has been to analyse three-dimensional models considering the risk factors related to length and diameter of dental implants, the ratio between anterior and posterior cantilevers [14], the dental occlusion with the distribution of load [15], the mesial cantilever structure [16], considerations of the material of the prosthetic structure [17], and bone tissue inflammation, resorption, and quality. Several biomedical investigations have used engineering to recreate models of medical devices in order to study the stress distribution after the medical tools are applied in situ. FEM (Finite Element Method) and FEA (Finite Element Analysis) have been widely applied in the simulation of the effects of tensions over the implant and its surrounding bone [1,7,9,11,12,13,14,15]. Specifically in the field of dentistry, the dynamic evaluation of prosthodontics components has been investigated in several published papers. However, comparing the data with the results of the present study, the stress of the peri-implant bone was due to the masticatory constant cycle. The data showed how the position and the length of the prosthodontics components like dental implant diameter or length of the cantilever strongly influence the tension and the bone resorption and remodelling [5,6,7,8,9,10].

The masticatory cycle is structured on vertical, transverse, and horizontal loads creating bending moments, which generate tension gradients in the dental fixtures and in the peri-implant bone tissue.

The dental implant’s clinical success and the survival rate have been recently investigated in numerous retrospective studies [16,17,18,19,20]. Several times, the anatomical condition of the jaws and the atrophic bone seems to be a limit for dental implant rehabilitation and for the long–term success of prosthodontics devices. As in the present study, correct planning before performing dental implant surgery is fundamental for the respect of important anatomical structures like sinus lift or mental nerve. In recent studies, authors concluded how the stress and the tension of the chewing cycle can be added as a main cause of prosthetics component fracture and of peri-implant bone resorption and loss. This transference is related to several parameters like the type of loading, the bone–implant connection, the length and diameter of the fixtures, the shape and the chemical features of the implant surface, the prosthodontics type, and the quantity and quality of the peri-implant bone tissue. Therefore, avoiding anatomical structure when placing dental implants with strong inclinations or degrees is a not a confident biomechanical solution and may lead the system to failure [14,15,16,17,18,19,20,21,22].

For this reason, engineering tools like FEA can predict the stress distribution of a system in which dental implant, bone tissue, and prosthodontics components are involved [18,19,20].

It has long been recognised that both implant and bone should be stressed within a limited range for physiologic homeostasis. Overload can cause bone resorption or fatigue fracture of the implant neck area, whereas no load over the bone can induce atrophy and subsequent bone loss [20,21,22,23,24,25,26,27].

Within the limitation of the model used, several animal models investigated how the load and the compression force may influence healing and bone remodeling: Hassler et al. [25], analysing the load cells in rabbit calvaria, underlined how the target compressive stress level for maximum bone growth occurs at 1.8 MPa, levelling off to a control level at 2.8 MPa; Skalak [26] demonstrated how the close apposition of bone to the titanium implant surface means that under loading, the interface can be considered as a unit with no relative motion—this condition is fundamental for the transmission of tension from the implant to the surrounding bone at all parts of the interface and so the load is a significant element of the osteointegration concept.

Considering the load distribution during the masticatory cycle, numerous bioengineering studies on dental implants have demonstrated that the main stress concentration is located in the cortical bone and in the upper area, “the neck”, of the implant. Otherwise, when the maximum stress concentration is located in the trabecular bone, it occurs around the apex of the implant. For this reason, it should be preferable to apply high-rigidity prostheses than low-elastic-moduli alloys for the overdenture framework because of the lower tensions at the bone–implant contact area on the loading side [27,28,29].

Stegariou et al. [29] demonstrated in an in vitro study that the stress in the bone–implant interface fixed on resin overdenture prostheses (acrylic or composite) was similar to that with gold alloys or ceramic ones. However, by performing a bioengineering mechanical analysis, Skalak [28] stated that the presence of a resilient element in an implant prosthesis superstructure would decrease the high load rates that happen when chewing unexpectedly on a hard object, suggesting then the use of acrylic resin teeth. Nevertheless, another study [30] demonstrated no significant differences in the force absorption quotients of gold, porcelain, or resin prostheses. As demonstrated in the data of the present study, wide fixture diameters offer better strength and tension distributions under load [28,29,30,31,32].

Engineering models have been applied to evidence that stresses in cortical bone can be reduced when related to the implant diameter enlarging under both vertical and lateral loads. However, Holmgren et al. [33] demonstrated that applying the largest dental implant diameters is not the best choice for better distributing the tensions to the peri-implant bone tissue; within limits related to the dental implant shape and design, an ideal dental implant size should replace the size of the missing tooth roots in order to replace the stress magnitudes at the bone–implant interface, simulating the ratio between tooth and root previously located in that area.

Wiskott and Belser [34] have observed that the loss of osteointegration can be influenced by the high pressure during the implant placement over the bone tissue. The absence of adequate biomechanical coupling between the load-bearing fixture surface and the surrounding bone can be the main cause of no osteointegration. The anatomical osseous processes are subjected to cyclic physiological phases of bone remodelling characterised by tissue resorption and formation. Hansson [35] evaluated dental implants with smooth necks placed all the way up to the bone ridge. This FEM research found that retention elements located at the implant neck showed a major decrease in peak interfacial shear stresses. Authors suggested that the shape of the abutment connection (conical or hexagonal) could influence the peri-implant bone remodelling.

Clinical investigations underlined how the prosthodontics components play a significant role in the long-term clinical success of the dental implant rehabilitation. Failures like gold screw and abutment screw fractures can occur, as well as gold cylinder, framework, and implant fractures. Cervino et al. recently investigated the stress of the abutment connection screw. The main reason for these failures is not easily found and it involves several parameters like cyclic fatigue, oral fluids, and varied chewing patterns and loads. Biomechanically, the shape and the length of the fixture abutment screw seem to be fundamental in the long-term clinical success. Therefore, screw joint integrity at the implant–abutment screw joint and abutment cylinder screw joint is essential for continued integrity of prosthetic components [5,6,7,8,9,10].

An increasing number of FEM biomedical and engineering studies focus on biomechanical failures involving the screw joint and on screw loosening phenomena [35,36,37,38]. The abutment screw loosening or fracture problem frequently affects dental implants and implant-supported prostheses. When a screw is fastened to fix the prosthesis, a tensile force (preload) is built up in the shank of the screw. This preload acts on the body of the screw passing through the threads. The preload range, according to the manufacture recommendations, is forced ranging between 25 Nmm to 40 Nmm. In this way, it generates a clamping force between the abutment and the dental implant. The screw elongates when subjected to tensile forces during tightening. The more elongation there is, the better the stability of the screw in place. Thus, screw design is fundamental for avoiding fracture and it should allow main torque to be fixed into the body of the dental implant. During the masticatory cycles, the continuous loading associated with unloading cycles [6,7,34,35,36,37,38,39,40] generates connection screw loosening and failure. Because the prosthesis framework splints multiple implants, tension dispersion is more difficult than with the single-tooth implant condition: the prosthesis can be loaded not by a single force but by multiple loads and in numerous angles [5,38].

Since the first studies on osteointegration, dental implants have been largely applied for the treatment of completely and partially edentulous patients [39,40,41,42,43]. Despite the high survival rates documented by several clinical investigations, early or late implant failures are still unpredictable and numerous papers have not focused on the difference between clinical success and dental implant survival rate [44]. Structural problems and failures have frequently been reported after prosthodontics treatment [45,46]. Usually, structural or biomechanical fractures are agreed to be significant particularly during the project of restorative treatments and the shaping of prosthetic appliances. For this reason, the present study aims to minimise the possibility of reducing prosthodontics and clinical problems highlighting the stress distribution and the masticatory cycles.

The oral cavity masticatory environment can be considered as a complex biomechanical system. Therefore, a large number of studies replacing the mechanical features of dental prosthetic devices for removable, fixed, and implant treatments have been conducted in vitro [47]. The use of engineering expertise in dentistry has helped the understanding of biomechanics aspects related to osteointegrated implants. However, still today the mechanisms responsible for implant non-integration, breaks, or fractures are not fully understood, and the international data about the influences of several biomechanical parameters are not conclusive [47].

The stress occurring around the implants is so important that in dentistry, many studies have reported methods to minimise this stress [48], and the present study results offer significant suggestions for placing dental implants where the stress is better distributed over prosthodontics components and mandibular bone.

The finite element method is a numerical procedure for analysing structures. Finite element analysis (FEA) is able to recreate a three-dimensional model by using algebraic equations solved by a digital computer [49]. FEA is a method for producing a solution to a difficult mechanical problem by dividing the system domain into a collection of much smaller and simpler domains in which the field variables can be interpolated with the use of shape functions [50]. In other words, FEA is a method wherein instead of seeking a solution function for the entire domain, one formulates the solution functions for its finite elements and combines them properly to obtain the solution to the whole structure, mixing elements of different types, shapes, and physical properties in a single computer program which allows the selection of program type, geometry, boundary conditions, element selection, and so on [50,51,52]. To define a good model obtained by FEA, experience and good engineering judgment are needed [53]. Tension distribution is influenced by numerous parameters like assumptions made in modelling geometry, material properties, boundary conditions, and the bone–implant interface. To realise more precise stress conditions, advanced digital imaging programs can be used to finally have the bone tissue geometry more similar to reality; the anisotropic and nonhomogeneous characteristics of the material have to be evaluated; moreover, the boundary conditions must be carefully treated with the use of computational recreating software. In addition, modelling of the bone–implant interface should incorporate the actual osteointegration contact area in cortical bone as well as the detailed trabecular bone contact pattern through the use of contact algorithms in FEA [53]. Such analysis allows us to change several conditions and allows us to measure the tension dispersion over the implants at optional points that are not easily clinically evaluated [54].

FEA has been used extensively in the prediction of biomechanical performance of dental implant systems, and of load distribution and stress located at the bone–implant interface. Several studies have concluded that the parameters that significantly condition load transfer at the bone–implant interface including the type of loading are the implant and prosthesis material properties, implant length and diameter, implant shape, structure of the implant surface, nature of the bone–implant interface, and the quality and quantity of the surrounding bone [55].

The mechanical features of the fixtures like length, diameter, and shape can be modified easily during the dental implant creation by software. The elastic moduli of the bone tissue related to the bone quality and quantity need to be evaluated before the treatment and should determine the implant choice. The clinical condition related to multiple-implant prosthesis design like an overdenture bar over dental implants can be recreated by FEA. The model has recreated boundary and extreme biomechanical situations when factors such as implant inclination, implant position, prosthetic material properties, superstructure beam design, cantilever length, bar system, bar span length and stiffener height, and overdenture attachment type are optimised [24,28,56,57].

Within the limitation of the present virtual model of investigation and considering all the different anatomical and physiological individual differences of each patient, the oral surgeon should always cooperate with the prosthodontics dentist in order to plan the correct dental implant rehabilitation for edentulous mandibular jaws considering the stress distribution over the prosthesis structure, over dental implant components, and over the bone area as well, related to the implant position. Data reported in the recent literature demonstrate how FEA is an engineering computational method able to reproduce and to simulate conditions close to the clinical dental implant biomechanics. Moreover, the present study offers the three-dimensional recreation of the clinical conditions realizing an overdenture bar, dental implant, and peri-implant bone tissue, aiming to help clinicians and practitioners in the choice of material avoiding bone resorption due to prosthodontics component overload [23,24,25,26,27,28,29,30,31,32,33,47,57].

The data reported from the present investigation underlines how the long-term success of the dental implant structure supporting an overdenture bar prosthesis is influenced by the dental implant position. 

## 5. Conclusions

According to the data obtained from this study, the stress seems to be better tolerated when the bar is loaded over 6 rather than over 4 dental implants. A wide diameter is suggested for the posterior area. Nevertheless, lower stress values on the prosthetic system may not justify the costs and work for two additional implants.

## Figures and Tables

**Table 1:** Mechanical characteristics of the different areas of mandibular bone and of titanium alloy of the screw [5,6,7,8,9,10].

No. Area	Cortical Bone			Spongy Bone		
	E (MPa)	G (MPa)	υ	E (MPa)	G (MPa)	υ
Zone 1	1.68 × 10^10^	6.24 × 10^9^	0.345	7.27 × 10^8^	2.71 × 10^8^	0.345
Zone 2	1.68 × 10^10^	2.34 × 10^9^	0.345	7.27 × 10^8^	2.71 × 10^8^	0.345
Zone 3	1.93 × 10^10^	7.41 × 10^9^	0.236	8.35 × 10^8^	3.37 × 10^8^	0.236
Zone 4	1.93 × 10^10^	7.41 × 10^9^	0.236	8.35 × 10^8^	3.37 × 10^8^	0.236
Zone 5	2.40 × 10^10^	9.71 × 10^9^	0.236	1.04 × 10^9^	4.21 × 10^8^	0.236
Zone 6	2.40 × 10^10^	9.71 × 10^9^	0.236	1.04 × 10^9^	4.21 × 10^8^	0.236
Zone 7	2.04 × 10^10^	8.25 × 10^9^	0.236	8.83 × 10^8^	3.57 × 10^8^	0.236
**Titanium Alloy (grade 4)**	**E (MPa)** = 1.05 × 10^11^		**G (MPa)** = 4.60 × 10^10^		**υ** = 0.33	

**Table 2:** Distances between fixtures placed in the mandible and cantilever lengths.

ZONE	Trial 01	Trial 02	Trial 03	Trial 04
a1	10 mm	13 mm	-	-
a2	6 mm	6 mm	6 mm	12 mm
a3	7.5 mm	7.5 mm	7.5 mm	7.5 mm
a4	6 mm	6 mm	6 mm	12 mm
a5	10 mm	13 mm	-	-
l1	8.5 mm	5.5 mm	18.5 mm	12.5 mm
l2	8.5 mm	5.5 mm	18.5 mm	12.5 mm

**Table 3:** Dimensions of the elements in the various components of the biomechanical system.

Bone Area			Prosthesis	
**Area**	**Cortical Bone**	**Spongy Bone**	**Screw**	0.5 mm
Zones 1,2	2 mm	2 mm		
Zones 3–7	1.5 mm	1.5 mm	**Cantilever**	0.8 mm
